# Simple and Ozonized Oil of Turpentine

**Published:** 1854-12

**Authors:** 


Simple and Ozonized Oil of Turpentine—Some very interesting experiments have been made by Dr. Seitz, on the comparative action of the simple and the ozonized oil of turpentine. The latter substance is thus prepared: The common oil of turpentine is placed in white bottles, of which it only fills the half or quarter, and is then freely exposed to sunlight. From time to time the bottle is opened, so that the atmospheric air may have free ingress. The oil thus prepared has the smell and taste of peppermint oil, smelling disagreeably, tasting hot and bitter, and giving to the tongue a peculiar pain and sensation of cold. From experiments on animals, it is found that, on the mucous membrane of the mouth and the digestive organs, the ozonized oil acts like the simple oil; it irritates, and causes an increased flow of saliva and mucus. It is then rapidly absorbed; the pulse increases in fullness and frequency, the respirations are quicker, and when large doses are given, they become even painfully so. If the doses are frequently repeated, inflammations of the endocardium and pericardium, and congestion and hæmoptoic infarctus of the lungs, are caused. Small doses act as excitants to the nervous system, but large doses cause stupor, convulsions, and paralysis. Like the simple, the ozonized oil passes off through the lungs and the kidneys, giving to the breath the smell of the oil, and to the urine the usual violet odour. The quantity of urine is increased. In one case, in a a horse, albumen, sugar, and benzoic acid appeared in it. In men the ozonized oil, in doses of five to fifteen drops, caused a sensation of coldness on the tongue, and a slight pricking sensation; the saliva was increased; there was a feeling of warmth in the stomach; the skin became hotter, and the pulse more frequent; the urine had the violet odour, but presented no other change. Applied to the skin, it produced the same effect as the non-oxygenized oil, such as redness and feeling of warmth. The ozonized oil has been given in various diseases with some good effect, viz:

in chronic cystitis and in incontinence of urine. In menorrhagia, and in cases of hæmatemesis, it was also useful. But its most decided action was evinced in cases of gouty and rheumatic pains, and especially in sciatica. The dose is from ten to twenty drops on sugar, or in sugar and water, or mixed with honey, or the yolk of an egg.—*Schmidt's Jahrb.*

